# HIV-1 Nef Enhances Dendritic Cell-Mediated Viral Transmission to CD4^+^ T Cells and Promotes T-Cell Activation

**DOI:** 10.1371/journal.pone.0034521

**Published:** 2012-03-30

**Authors:** Corine St. Gelais, Christopher M. Coleman, Jian-Hua Wang, Li Wu

**Affiliations:** 1 Center for Retrovirus Research, Department of Veterinary Biosciences, The Ohio State University, Columbus, Ohio, United States of America; 2 Key Laboratory of Molecular Virology and Immunology, Institute Pasteur of Shanghai, Shanghai Institutes for Biological Sciences, Chinese Academy of Sciences, Shanghai, China; 3 Department of Microbial Infection and Immunity, The Ohio State University Medical Center, Columbus, Ohio, United States of America; George Mason University, United States of America

## Abstract

HIV-1 Nef enhances dendritic cell (DC)-mediated viral transmission to CD4^+^ T cells, but the underlying mechanism is not fully understood. It is also unknown whether HIV-1 infected DCs play a role in activating CD4^+^ T cells and enhancing DC-mediated viral transmission. Here we investigated the role of HIV-1 Nef in DC-mediated viral transmission and HIV-1 infection of primary CD4^+^ T cells using wild-type HIV-1 and Nef-mutated viruses. We show that HIV-1 Nef facilitated DC-mediated viral transmission to activated CD4^+^ T cells. HIV-1 expressing wild-type Nef enhanced the activation and proliferation of primary resting CD4^+^ T cells. However, when co-cultured with HIV-1-infected autologous DCs, there was no significant trend for infection- or Nef-dependent proliferation of resting CD4^+^ T cells. Our results suggest an important role of Nef in DC-mediated transmission of HIV-1 to activated CD4^+^ T cells and in the activation and proliferation of resting CD4^+^ T cells, which likely contribute to viral pathogenesis.

## Introduction

Dendritic cells (DCs) are among the first cells that encounter HIV-1 at the mucosa, and play a critical role in HIV-1 infection [1], [2], [3], [4]. Immature DCs allow productive HIV-1 replication and long-term viral dissemination [5], [6], [7], [8], [9]. DC-SIGN (DC-specific intercellular adhesion molecule 3 grabbing non-integrin) is a C-type lectin that enhances HIV-1 *trans*-infection [10]. DC-SIGN-expressing immature DCs from human rectal mucosa efficiently bind and transfer HIV-1 to CD4^+^ T cells [11]. Although DC-SIGN^+^ cells comprise only 1–5% of total mucosal mononuclear cells, they account for greater than 90% of HIV-1 binding, since DC-SIGN antibodies block 90% of this binding [11]. Previous studies have suggested that DC-SIGN partially contributes to immature DC-mediated HIV-1 transmission and that DC maturation enhances HIV-1 transmission efficiency [12], [13], [14], [15], [16], [17], [18], [19], [20], [21], [22]. However, the precise mechanisms underlying DC-mediated HIV-1 transmission to CD4^+^ T cell remain to be defined.

The Nef protein of HIV-1 and simian immunodeficiency virus (SIV) is a pathogenic factor *in vivo* [23], [24], [25], [26], and contributes to many aspects of primate lentiviral immunopathogenesis through its interactions with host proteins [27], [28], [29], [30], [31]. HIV-1 Nef is required for efficient viral replication in co-cultures of DCs and activated CD4^+^ T cells or peripheral blood mononuclear cells (PBMCs) [9], [32], [33]. HIV-1 Nef up-regulates DC-SIGN expression of infected DCs, increases clustering of DCs with CD4^+^ T cells, and modulates DC and CD4^+^ T cell activation [9], [34], [35], [36]. Immunoactivation facilitates HIV-1 replication and leads to CD4^+^ T cell depletion and AIDS pathogenesis [37], but it is unclear whether Nef expression in HIV-1 infected DCs plays a role in activating CD4^+^ T cells and enhancing cell-to-cell transmission. Previous studies have indicated inconsistent functions of HIV-1 Nef in CD4^+^ T cell activation [29], which need to be clarified using wild-type (WT) HIV-1 and Nef-mutated viruses of primary resting CD4^+^ T cells. Moreover, HIV-1 Nef down-regulates surface CD4 expression on infected DCs and CD4^+^ T cells [9], [38]. We have reported that CD4 co-expression with DC-SIGN blocks DC-SIGN-mediated transmission of HIV-1 [9]. However, it remains unclear whether the effects of Nef on the expression of CD4 and DC-SIGN affect DC-mediated HIV-1 transmission to CD4^+^ T cells.

In this study, we investigated the role of HIV-1 Nef in DC-mediated viral transmission and HIV-1 infection of primary CD4^+^ T cells using WT HIV-1 and HIV-1 expressing mutated Nef proteins. We show that Nef promotes DC-mediated HIV-1 transmission to activated CD4^+^ T cells, and that Nef expression promotes activation and proliferation of resting CD4^+^ T cells to enhance HIV-1 infection of these cells. Our results suggest an important role of Nef in DC-mediated transmission of HIV-1 to activated CD4^+^ T cells and in the activation and proliferation of resting CD4^+^ T cells, which likely contribute to viral pathogenesis.

## Results

### HIV-1 Nef enhances DC-mediated HIV-1 transmission to CD4^+^ T cells and modulates CD4 expression of DCs

**Figure 1:**
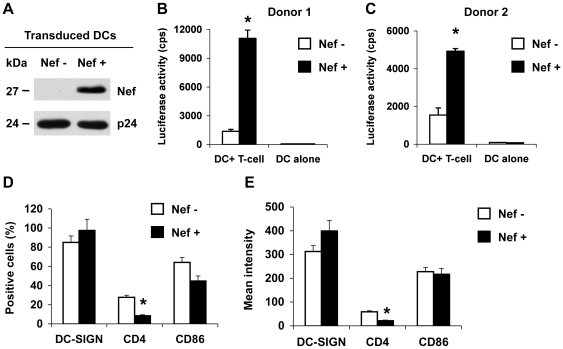
HIV-1 Nef enhances DC-mediated HIV-1 transmission to Hut/CCR5 T cells and modulates CD4 expression of DCs. Immature DCs were generated from purified monocytes by treatment with GM-CSF and IL-4 for 5 days. Immature DCs were transduced with a Nef-expressing lentiviral vector (Nef+) or a negative control vector (Nef−). (A) Nef expression in vector-transduced DCs was detected at five days post-transduction by immunoblotting. HIV-1 p24 was used a positive control. (B and C) Nef expression in DCs promotes HIV-1 transmission. DC-mediated transfer of single-cycle, Nef-defective, R5-tropic luciferase HIV-1 to CD4^+^ Hut/CCR5 cells was measured at 2 days post-infection (dpi). The data show the mean ± SD of triplicate samples of cells from two different donors. (A–C) One representative experiment out of four is shown. cps, counts per second. (D and E) Transduced DCs were analyzed by flow cytometry for the positive percentage (D) and the mean intensity (E) of DC-SIGN, CD4 and CD86 expression at 5 days post-transduction. The data show the mean ± SD of three independent experiments. (B–E) *, Significant differences compared with the Nef-negative controls (*P*<0.05).

To examine whether Nef expression in DCs can modulate DC-mediated HIV-1 transmission to CD4^+^ T cells, immature monocyte-derived DCs (MDDCs) were transduced with a vesicular stomatitis virus G protein (VSV-G)-pseudotyped, Nef-expressing lentiviral vector and then tested for the ability to transmit single-cycle HIV-1 to CD4^+^ Hut/CCR5 T cells. A VSV-G-pseudotyped Nef-deleted lentiviral vector was used as a negative control. These HIV-1-derived lentiviral vectors are defective for *vif*, *vpr*, *vpu*, and *env* genes [39]. Nef expression in the transduced MDDCs was confirmed by immunoblotting (Fig. 1A). The vector-transduced cells were pulsed with R5-tropic, Nef-defective, single-cycle luciferase HIV-1 [12], and co-cultured with CD4^+^ Hut/CCR5 T cells. Nef expression in DCs enhanced HIV-1 transmission by 6- to 8-fold (*P*<0.01) relative to the control vector-transduced DCs (Fig. 1B and 1C). These results indicate that Nef expression in DCs can promote DC-mediated HIV-1 transmission to CD4^+^ T cells.

To examine the effect of HIV-1 Nef expression on DC-SIGN and CD4 levels and DC maturation of the infected DCs, cell-surface DC-SIGN, CD4 and the DC maturation marker CD86 were measured by flow cytometry. Nef expression in transduced DCs significantly decreased CD4 expression by at least 43% (Fig. 1D and 1E). The expression of DC-SIGN and CD86 was not significantly modulated by Nef expression (Fig. 1D and 1E), suggesting DC maturation was not affected by Nef expression in transduced DCs. Thus, Nef expression in MDDCs can efficiently enhance HIV-1 transmission and down-regulate CD4 expression.

### Effects of Nef on HIV-1 infection and maturation of DCs

Our previous studies using replication-competent HIV-1 suggested that Nef facilitates DC-mediated viral transmission to co-cultured CD4^+^ T cells [40]. To better understand Nef-dependent enhancement of HIV transmission by DCs, replication-competent HIV-1 expressing mutated Nef proteins were compared with WT HIV-1 for their effects on HIV-1 infection and maturation of DCs during a time-course up to 7 days post-infection (dpi). Mutation of the Nef myristoylation motif (glycine to arginine, G2A) or a conserved dileucine motif [L164L/AA (LL/AA)] confers distinct defects on Nef function [41]. The G2A mutant blocks Nef localization to the plasma membrane and the LL/AA mutant disrupts Nef interactions with the clathrin endocytosis machinery [30], [41]. R5-tropic HIV-1_NLAD8_ and derived Nef-inactivated virus were used because DCs are more susceptible to R5 HIV-1 than to X4 HIV-1 infection [40], [42].

**Figure 2:**
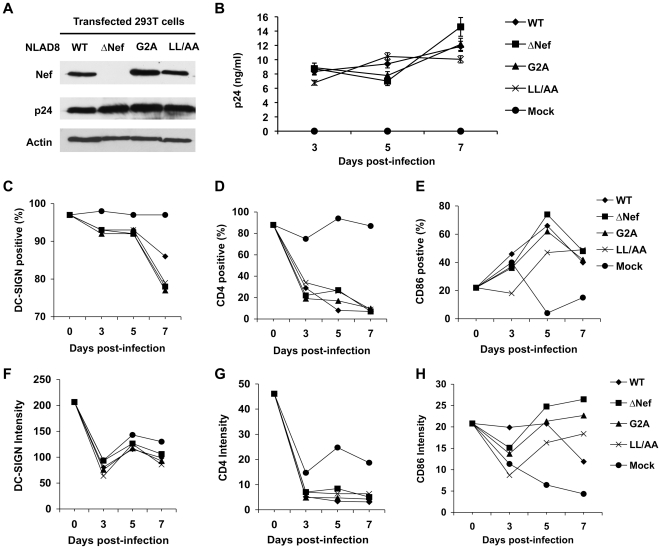
Effects of Nef on HIV-1 infection and maturation of DCs. (A) Nef expression of wild-type (WT) HIV-1_NLAD8_ and Nef-mutated viruses (ΔNef, G2A, and LL/AA). HEK293T cells transfected with proviral DNA were subjected to immunoblotting at two days post-transfection. HIV-1 p24 was used as a positive control for viral protein expression and actin was a loading control. (B) Comparable infection of DCs with HIV-1_NLAD8_ WT and Nef-mutated viruses. Immature DCs were generated from purified monocytes by treatment with GM-CSF and IL-4 for 5 days. DCs were infected with HIV-1 for 16 h and cells were washed thoroughly and cultured for the indicated time course. Cell-free supernatants from the infected DCs were measured for HIV-1 p24 by ELISA at the indicated times. Error bars represent the standard deviation of the mean of triplicate samples. HIV-1 infected DCs were analyzed by flow cytometry for the positive percentage and the mean intensity of DC-SIGN (C and F), CD4 (D and G) and CD86 expression (E and H). The data shown represents one of three independent experiments using cells from three different donors.

The Nef expression of these viruses was confirmed by immunoblotting (Fig. 2A). HIV-1 infection of DCs was comparable between WT HIV-1 and the Nef-mutated viruses when p24 production was measured by enzyme-linked immunosorbent assay (ELISA) (Fig. 2B). Compared with the mock-infected DCs, HIV-1 infected DCs showed decreased DC-SIGN surface levels over the time course of infection. At 7 dpi, WT HIV-1 infected DCs maintained higher levels of DC-SIGN relative to DCs infected with Nef-mutated viruses [Fig. 2C (*P*<0.05) and 2F]. Consistent with our previous results [9], WT HIV-1 infection efficiently decreased (*P*<0.05) surface CD4 expression in DCs relative to DCs infected with Nef-mutated viruses at 5 dpi (Fig. 2D). Surface CD4 levels of HIV-1 infected DCs were significantly decreased (*P*<0.01) compared with mock-infected DCs and no significant difference (*P*>0.05) between WT HIV-1 and Nef-mutated viruses was observed at 7 dpi (Fig. 2D and 2G), perhaps because WT HIV-1 and Nef-mutated viruses encode functional Vpu, which can also efficiently down-regulate CD4 expression [43]. Surface CD86 expression in HIV-1 infected DCs increased at 5 and 7 dpi compared with that of mock-infected DCs (Fig. 2E and 2H), suggesting that HIV-1 infection induced DC maturation. The CD86 levels of DCs infected with ΔNef HIV-1 were similar to or slightly higher than those infected with WT and other Nef-mutated viruses (Fig. 2E and 2H). Similar results were obtained when DCs from two additional donors were examined. Together, our results suggest that replication-competent HIV-1 infection of DCs can down-regulate DC-SIGN and CD4 expression and facilitate DC maturation in a largely Nef-independent manner.

### Nef enhances HIV-1 infection of activated peripheral blood lymphocytes (PBLs) and facilitates DC-mediated HIV-1 transmission to activated autologous PBLs

**Figure 3:**
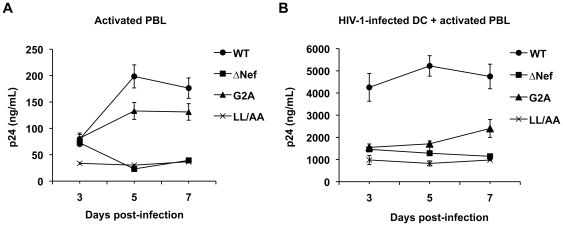
Nef enhances HIV-1 infection of activated PBLs and DC-mediated HIV-1 transmission to activated autologous PBLs. (A) Nef enhances HIV-1 replication in activated PBLs. HIV p24 in the cell supernatants of DCs infected with WT HIV-1_NLAD8_ or Nef-mutated viruses was measured by ELISA. (B) Nef promotes DC-mediated HIV-1 transmission to activated PBLs. Immature DCs were generated from purified monocytes by treatment with GM-CSF and IL-4 for 5 days. DCs infected with WT HIV-1_NLAD8_ or Nef-mutated viruses were co-cultured with PHA-activated autologous PBLs and HIV p24 in the cell supernatants was measured. (A and B) The data show the mean ± SD of triplicate samples. The data shown represents one of four independent experiments using cells from four different donors.

To examine the effects of HIV-1 Nef expression on viral replication and transmission in a physiologically relevant system, HIV-1 infection of activated PBLs and DC-mediated HIV-1 transmission to autologous PBLs was compared using the WT HIV-1 and Nef-mutated viruses. Compared with WT HIV-1, the replication of ΔNef and Nef (LL/AA) HIV-1 was 4- to 5-fold lower in activated PBLs, while the replication of Nef (G2A) mutant was 1.4-fold lower at 5 and 7 dpi (Fig. 3A). All three Nef-mutated viruses showed impaired DC-mediated HIV-1 transmission to activated PBLs, which was 2- to 4-fold lower (*P*<0.05) relative to WT HIV-1 infection (Fig. 3B). At 3 dpi, although comparable HIV-1 replication of WT HIV-1 and Nef-mutated viruses was observed in activated PBLs (Fig. 3A), WT HIV-1 infected DCs enhanced viral spread 3-fold more efficiently relative to Nef-mutated viruses in DC-PBL cocultures (Fig. 3B). These results suggest that Nef is important for efficient HIV-1 replication in activated PBLs and DC-mediated HIV-1 transmission to activated PBLs.

### HIV-1 Nef activates primary resting CD4^+^ T cells, resulting in increased viral infection and T-cell proliferation

**Figure 4:**
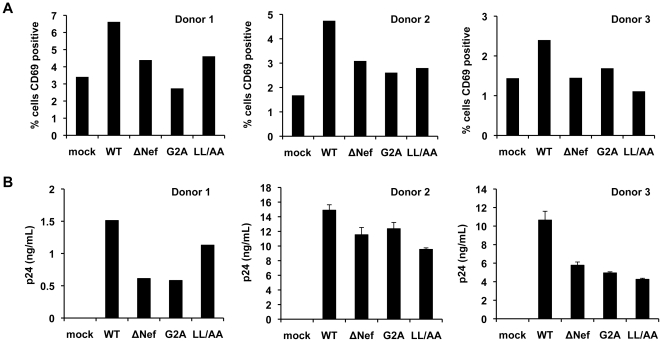
HIV-1 Nef activates primary resting CD4^+^ T cells, resulting in increased T-cell proliferation and viral infection. (A) HIV-1 Nef increases the expression of the transient T-cell activation marker CD69 in resting CD4^+^ T cells infected with replication-competent WT or Nef-mutated HIV-1 at 3 dpi. Mock-infected cells were used as background controls for CD69 staining and flow cytometry. Resting CD4^+^ T cells were isolated from PBMCs using immunomagnetic particles and were cultured in the presence of IL-2 before and during HIV-1 infection. Resting CD4^+^ T cells were cultured for 8 days, infected with 5 ng p24 of HIV-1 or a media control for 2 hours, washed and then cultured for an additional 3 days. The data shown represents three independent experiments using cells from three different donors. (B) WT Nef increases p24 production in the supernatants of HIV-1-infected resting CD4^+^ T cells at 3 dpi. Mock-infected cells were used as negative controls (undetectable p24). Data from three different donors are shown, which are matched to those in (A). Data displayed are the mean ± SEM of duplicate samples for each infection except the results of donor 1, which show the p24 values from a single sample.

In the early stages of HIV-1 infection, the majority of CD4^+^ T cells are naïve and resting, and can be latently infected by HIV-1 [44], [45]. Previous studies have indicated that wild-type Nef plays an important role in HIV-1 infection and activation of CD4^+^ T cells [46], [47]. To better understand the role of Nef in HIV-1 infection of resting CD4^+^ T cells, we investigated the effect of WT HIV-1 and Nef-mutated viruses on the activation of resting CD4^+^ T cells and viral replication using cells from three different donors. The expression levels of CD69, an early marker of CD4^+^ T cell activation [48], in WT HIV-1 infected cells were increased compared to mock-infected control cells at 3 dpi (Fig. 4A), indicating that HIV-1 infection activates resting CD4^+^ T cells. By contrast, all Nef-mutated viruses showed reduced or baseline levels of CD69 expression compared to WT HIV-1-infected cells (Fig. 4A), suggesting that functional Nef protein is required for the activation of resting CD4^+^ T cells. This trend correlated with the levels of p24 in the supernatants, with the WT infected cells showing increased p24 production over the Nef-mutated viruses at 3 dpi (Fig. 4B).

**Figure 5:**
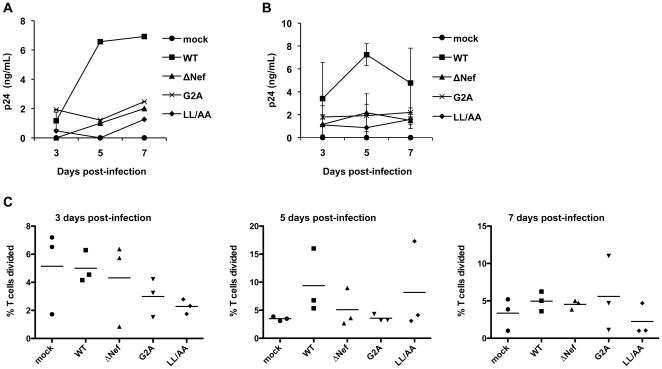
Nef promotion of HIV-1 replication in primary resting CD4^+^ T cells is associated with T cell proliferation. Resting CD4^+^ T cells were isolated from PBMCs using immunomagnetic particles and were cultured in the presence of IL-2 before and during HIV-1 infection. (A) Resting CD4**^+^** T cells were cultured for 8 days and then infected with 5 ng p24 of replication-competent WT or Nef-mutated HIV-1. HIV-1 p24 production in the supernatants was measured at the times indicated. Representative data from one donor are shown. (B) Resting CD4**^+^** T cells were cultured for 8 days and then infected with 5 ng p24 of WT HIV-1 or Nef-mutated viruses. HIV-1 p24 production in the supernatant was measured at the times indicated. Mean values of two independent experiments on different donors are shown. (C) CFSE-labeled resting CD4**^+^** T cells (cultured for 8 days in the presence of IL-2) were mock infected or infected with 5 ng p24 of WT or Nef-mutated HIV-1. CD4**^+^** T cell proliferation was measured by flow cytometry at the indicated times. Data represent three independent experiments using cells from three different donors. Each symbol in the plots represents a single experiment result and the horizontal bars in the plots indicate the mean values of three independent experiments. There is no statistically significant difference in T-cell proliferation among WT and Nef-mutated HIV-1 infected CD4^+^ T cells.

CD69 is a transient phenotypic marker of CD4^+^ T cell activation [49]. To functionally assess the effect of Nef expression on resting CD4^+^ T cell activation, we performed a flow cytometry based T-cell proliferation assay using carboxyfluorescein diacetate succinimidyl ester (CFSE) labeling and also measured p24 production over a period of 7 days of infection. We assessed p24 production by HIV-1-infected resting CD4^+^ T cells from two healthy donors. The p24 levels in the supernatants of WT HIV-1-infected resting CD4^+^ T cells were 6-fold higher (5 and 7 dpi, *P*<0.02) than those of Nef-mutated HIV-1 infected T cells (Fig. 5A). A similar trend was observed when the average results of two donors were combined (Fig. 5B), suggesting that HIV-1 replication in resting CD4^+^ T cells occurs in a Nef-dependent manner. Furthermore, the reverse transcriptase inhibitor azidothymidine was able to completely block p24 production in the supernatants, confirming productive HIV-1 replication in resting CD4^+^ cells (data not shown).

We then investigated whether the effect of Nef on HIV-1 infection of resting CD4^+^ T cells correlated with Nef-dependent T-cell proliferation. To evaluate the overall effect of Nef expression on proliferation in resting CD4^+^ T cells, data derived from three different donors' cells were combined (Fig. 5C). Whilst there was large donor-to-donor variability, WT HIV-1 infected CD4^+^ T cells showed consistently higher T-cell proliferation at 5 dpi than mock-infected and Nef-mutated HIV-1 infected cells (Fig. 5C). However, there was no statistically significant difference in T-cell proliferation among WT HIV-1 and Nef-mutated HIV-1 infected CD4^+^ T cells (Fig. 5C).

**Figure 6:**
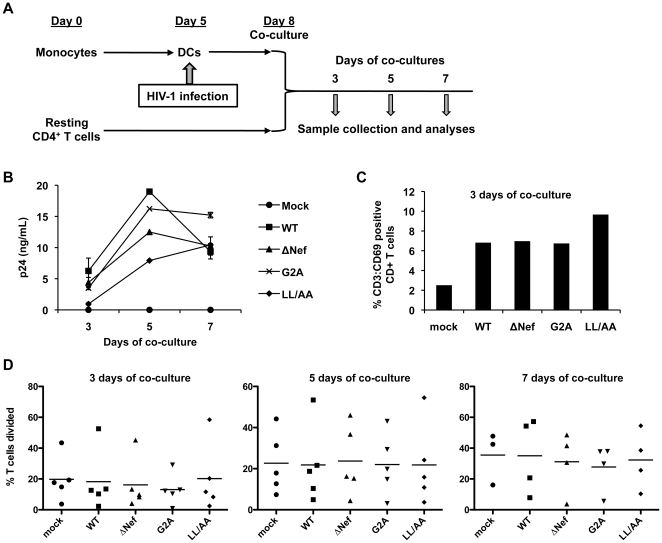
HIV-1-infected DCs cannot significantly increase resting CD4^+^ T cell proliferation in DC-T cell co-cultures. (A) Schematic representation of the experiment procedures. Primary MDDCs and autologous resting CD4^+^ T cells were used. (B) DCs infected with replication-competent WT or Nef-mutated HIV-1 were co-cultured with uninfected resting CD4^+^ T cells and HIV-1 p24 production in the supernatants was measured at the indicated times of co-cultures. Immature DCs were generated from purified monocytes by treatment with GM-CSF and IL-4 for 5 days. Resting CD4^+^ T cells were isolated from PBMCs using immunomagnetic particles and were cultured in the presence of IL-2 for 8 days. (C) Co-culture with HIV-1-infected DCs increases the surface expression of the transient T-cell activation marker CD69 on resting CD4^+^ T cells. CD69 expression was assessed at 3 days of co-cultures by flow cytometry. Mock-infected cells were used as background controls. These data are donor matched to Figure 6B. (D) CD4^+^ T cell proliferation in the co-cultures of HIV-1 infected DCs. DCs were mock infected or infected with WT or Nef-mutated HIV-1 for 3 days prior to co-culture with CFSE-labeled, uninfected resting CD4^+^ T cells. T cell proliferation was measured by flow cytometry at the indicated times of co-cultures. Data represent five independent experiments using cells from five different donors. Each symbol in the plots represents a single experiment result and the horizontal bars in the plots indicate the mean values of five independent experiments.

An increase in proliferation over time is consistent with active T cell proliferation and survival of each subsequent generation, whilst decreases in T cell proliferation are likely due to activation induced CD4^+^ T cell apoptosis or HIV-1-induced T cell death. These results suggest that Nef facilitates resting CD4^+^ T cell activation and proliferation, thus providing an environment to facilitate HIV-1 replication. It is also important to consider that the resting CD4^+^ T cells used in all of these experiments (Fig. 4, 5, 6) were in culture for 8 days in the presence of IL-2 (see experiment procedures indicated in Fig. 6A); therefore, it is possible that there is a degree of background activation in the resting CD4^+^ T cells. The decreases in the percentage of T cells divided over time (Fig. 5C mock infected controls) were likely due to activation-induced T cell death. Together, these results indicate that Nef enhances the activation and proliferation of HIV-1-infected resting CD4^+^ T cells and viral replication in these cells.

### HIV-1-infected DCs cannot significantly increase resting CD4^+^ T cell proliferation in DC-T cell co-cultures

To understand the potential role of Nef in the co-cultures of HIV-1-infected DCs and CD4^+^ T cells, we next investigated the effect of Nef expression in DCs on the activation of co-cultured resting CD4^+^ T cells and on HIV-1 production. MDDCs were infected with HIV-1 and the Nef-mutated viruses for 3 days to allow productive infection to occur. At this point, autologous resting CD4^+^ T cells that had been cultured in media containing interleukin-2 for the duration of the DC generation were added to HIV-1-infected DCs at a cell number ratio of 1∶1. Co-cultured cells and the culture supernatants were collected and analyzed 3, 5, and 7 days later (Fig. 6A).

WT HIV-1 appeared to replicate more efficiently than all of the Nef-mutated viruses at 3 and 5 days of co-culture, as assessed by p24 production in the supernatants (Fig. 6B). For instance, significant enhancement in WT HIV-1 replication observed was a 6.6-fold and a 2.4-fold increase in p24 production over the Nef (LL/AA) mutant at 3 and 5 days of co-cultures, respectively (Fig. 6B), which were reproducible across three different donors. Moreover, azidothymidine was able to completely block p24 production in the supernatants, confirming productive HIV-1 replication in co-cultures (data not shown).

To assess the role of CD4^+^ T cell activation and proliferation in the enhancement of HIV-1 production, CD69 staining and T-cell proliferation assays were performed. To differentiate the T cells from the DCs in the co-cultures, cells were additionally stained for CD3, a general marker for T cells. At 3 days of co-culture, there was a 2- to 3-fold increase of CD69 expression by CD3^+^ T cells in co-culture with HIV-1-infected DCs infected over the mock-infected controls (Fig. 6C), while there were no significant differences between WT HIV-1 and Nef-mutated viruses infected cells (Fig. 6C). These data were also reproducible across three different donors (not shown). When the T-cell proliferation data from DC-T-cell co-cultures of five different donors were combined, there was no consistent trend for Nef-dependent proliferation of CD4^+^ T cells (Fig. 6D). There was no statistically significant difference in T-cell proliferation among WT HIV-1 and Nef-mutated viruses infected CD4^+^ T cells (Fig. 6D). These results suggest that Nef-mediated enhancement of HIV-1 replication in DC-T-cell co-cultures is not directly attributable to CD4^+^ T cell activation and proliferation.

## Discussion

In addition to DC-mediated HIV-1 *trans*-infection, long-term viral transfer to CD4^+^ T cells by DCs depends on HIV-1 production from infected DCs [6], [7], [50], [51]. Thus, we examined Nef modulation of DC-mediated HIV-1 transmission through *de novo* production of virus. WT and Nef-mutated HIV-1 replicated with similar kinetics in immature MDDCs. Nef expressing HIV-1-infected DCs promoted viral transmission to co-cultured CD4^+^ T cells. Nef modulation of DC-SIGN and CD4 expression was observed, though levels of DC-SIGN upregulation were limited in lentiviral vector transduced DCs. Our time-course analysis of DCs infected with replication-competent WT HIV-1 and Nef-mutated viruses suggest that HIV-1 infection of DCs can down-regulate DC-SIGN and CD4 expression in a largely Nef-independent manner. By contrast, a previous study using immunofluorescence microscopy showed that DC-SIGN surface levels are upregulated in HIV-1-infected DCs at 4 dpi in a Nef-dependent manner, which increases clustering of DCs with T lymphocytes and HIV-1 transmission [34]. Although different experimental approaches might result in the discrepancy of the results, Nef-mediated DC-SIGN upregulation may not fully explain DC-enhanced HIV-1 transmission to CD4^+^ T cells. Previous studies indicated that DC-SIGN only partially accounts for or plays very little role in DC-mediated HIV-1 transmission [12], [20], [52]. Of note, differential modulation of CD86 and DC-SIGN expression in DCs was observed between lentiviral transduction and WT HIV-1 infection (Fig. 1 and 2). The data in Fig. 1 represent a situation in which Nef was expressed in *trans*, in the absence of other HIV-1 accessory proteins. The results in Fig. 2 provide a model for what could occur during WT HIV-1 infection, with all HIV-1 accessory genes intact.

HIV-1 transmission efficiency can be enhanced by maturation of DCs [22], [53]. To exclude the possibility that Nef-promoted HIV-1 transmission results from Nef-induced DC maturation, the maturation marker CD86 was compared between WT and ΔNef HIV-1-infected DCs, and no Nef-induced upregulation of the maturation marker was observed. Similarly, adenovirus vector-expressed HIV-1 Nef in immature DCs triggers cytokine and chemokine production, and Nef-expressing DCs stimulate CD4^+^ T cell activation; but without up-regulation of DC maturation markers [36]. Thus, Nef may facilitate DC-mediated HIV-1 transmission by promoting DC-T cell interactions.

Nef-mediated suppression of T cell activation is a critical feature of non-pathogenic primate lentiviruses and HIV-2, whereas HIV-1 Nef induces T cell activation and contributes to viral pathogenicity [54]. In addition to the modulation of CD4 and DC-SIGN expression, HIV-1 Nef also down-regulates cell surface expression of MHC class I, CCR5 and CD28 [55], [56], [57], but upregulates surface expression of the invariant chain of MHC class II, TNF and the related LIGHT cytokines [58], [59]. Although there is no evidence that these modulations, other than CD4 and DC-SIGN expression, can affect DC-mediated HIV-1 transmission, Nef-mediated modulation of T cell function also contributes to viral transmission *in vivo*.

HIV-1 Nef plays a multifaceted role in viral infection and pathogenesis [29]. It has been previously shown that deletion of Nef and specific mutations in important Nef motifs, including the Nef (G2A) and Nef (LL/AA) mutants utilized in these experiments, causes attenuated HIV-1 replication in activated CD4^+^ T cells [60]. Previous studies have also suggested that Nef stimulates HIV-1 replication in primary CD4^+^ T cells by enhancing virion-associated gp120 levels of infected cells [61]. Moreover, Nef stimulates HIV-1 proviral DNA synthesis [62], and enhances HIV-1 infectivity resulting from inter-virion fusion [63]. It has been shown that Nef can be transferred from HIV-1-infected CD4^+^ cells to bystander cells upon cell-to-cell contact and contribute to the detrimental effect on bystander cells in viral infection [64]. Here we demonstrate that during direct infection of resting CD4^+^ T cells, HIV-1 is capable of causing activation and proliferation of resting CD4^+^ T cells *in vitro*, while deletion of Nef and mutations within Nef can reduce this effect. Enhanced infection of CD4^+^ T cells by the WT HIV-1 was observed by p24 quantification, indicating that HIV-1 is capable of facilitating its own replication by causing resting CD4^+^ T cell activation and proliferation, and that functional Nef protein plays a significant role in this activation process.

In DC-T-cell co-culture experiments, Nef expression by infected DCs did not appear to significantly enhance CD69 expression or proliferation of co-cultured CD4^+^ T cells above background activation caused by the co-culture itself. Nevertheless, Nef-mutated HIV-1 do not replicate as efficiently in these co-cultures as WT HIV-1 and are transmitted less efficiently than WT HIV-1. DCs are refractory to productive HIV-1 infection [3], which may explain the lack of significant activation of CD4^+^ T cells in cocultures. HIV-1 can efficiently induce DC maturation and activation of cocultured CD4^+^ T cells when the HIV-1 restriction in DCs is circumvented by SIV Vpx [65]. Recent studies have identified SAMHD1 as the DC- and myeloid-cell-specific HIV-1 restriction factor, which can be antagonized by Vpx proteins from sooty mangabey SIV or HIV-2 [66], [67]. Further investigation of the mechanisms underlying SAMHD1-mediated HIV-1 restriction in DCs and myeloid cells can aid in the understanding of viral pathogenesis [68].

In summary, we demonstrated that HIV-1 Nef enhances DC-mediated HIV-1 transmission to activated CD4^+^ T cells. HIV-1 Nef contributes to the activation and proliferation of infected resting CD4^+^ T cells. By contrast, Nef expression in infected DCs does not significantly affect DC-mediated CD4^+^ T cell activation or proliferation. These results provide new information in understanding the function of Nef as a viral pathogenic factor.

## Materials and Methods

### Plasmids

HIV-1 proviral vector pNL-Luc-E^−^R^−^ contains a firefly luciferase reporter gene [69]. The R5-tropic HIV-1_JRFL_ envelope (Env) expression plasmid pJRFL has been previously described [9]. HIV-1-based expression vectors pH 131 and pH 132 were kind gifts from Dr. Derya Unutmaz (New York University). These vectors with *vif*, *vpr*, *vpu*, and *env* deletions were derived from the HIV-1-eGFP construct [39]. The control vector, pH 131, has the encephalomyocarditis virus internal ribosomal entry site (IRES) and mouse heat stable antigen (HSA) in place of *nef*, pH 132 has a nef-IRES-HSA cassette and expresses full-length WT HIV-1 Nef. WT HIV-1 proviral vector pNLAD8 (R5-tropic) was a kind gift from Eric Freed [70] (National Cancer Institute-Frederick). HIV-1 *nef*-inactivated proviral vector pNLAD8ΔNef was a kind gift from Olivier Schwartz (Pasteur Institute). Proviral DNA expressing Nef (G2A) and Nef (LL/AA) mutants in the pNLAD8 backbone were generated as described [32] and confirmed by DNA sequencing.

### Cell culture

Human PBMCs were isolated from the buffy coat of healthy donors (American Red Cross Blood Service, Columbus, Ohio) as previously described [9]. Human primary CD14^+^ monocytes and CD4^+^ T cells were isolated from PBMCs using gradient centrifugation and immunomagnetic particles as described [9]. Immature DCs were generated from purified monocytes by treatment with granulocyte-macrophage colony-stimulating factor (GM-CSF) and interleukin 4 (IL-4) (50 ng/ml, R&D Systems) for 5 days as described [71]. Primary resting CD4^+^ T cells were cultured in the presence of 20 IU/ml of recombinant interleukin-2 (IL-2) (the NIH AIDS Research and Reference Reagent Program) and activated by phytohemagglutinin (PHA, 5 µg/ml) for 2 days as previously described [9]. PHA-activated PBLs were generated as previously described [9]. DCs and CD4^+^ T cells were more than 98% pure by flow cytometry analysis of surface markers as described [5]. Human embryonic kidney cell line HEK293T, human T cell line Hut/CCR5, and HIV-1 indicator cell line GHOST/X4/R5 (kind gifts from Dr. Vineet KewalRamani, National Cancer Institute, Frederick, Maryland, USA) have been previously described [20], [71], [72].

### HIV-1 stocks

The single-cycle infectious HIV-1 stocks were generated by calcium phosphate cotransfection of HEK293T cells with the pNL-Luc-E^−^R^−^ and an expression plasmid for HIV-1_JRFL_ Env as described [20]. Replication-competent WT and Nef-mutated HIV-1 stocks were generated separately by transfection of HEK293T cells [20] with proviral vectors pNLAD8, pNLAD8ΔNef, pNLAD8Nef (LL/AA), or pNLAD8Nef (G2A) as described [9]. All virus stocks were harvested 2 days post-transfection. The infectious units of virus stocks were evaluated by limiting dilution on GHOST/X4/R5 cells [20] as described [20]. HIV-1 p24 concentrations of viral stocks were measured by ELISA (SAIC-Frederick) as described [5].

### Cell transduction with the H131 and H132 vectors

Nef-expressing vector H132 and the control vector H131 were generated by co-transfection of HEK293T cells with pH 132 or pH 131 and the VSV-G expressing construct pVSV-G as described [20]. Immature DCs (2×10^6^) were incubated separately with VSV-G-pesudotyped H131 and H132 vectors for 2 hours in the presence of DEAE-dextran (10 µg/ml) as described [9]. Transduced DCs were washed and cultured for 5 days before immunoblotting and flow cytometry analyses.

### Immunoblotting

The H131 and H132 vector-transduced DCs and HEK293T cells that were transfected separately with pH 131, pH 132, or HIV-1 proviral DNA were lysed with 1% Triton X-100 supplemented with the presence of the protease inhibitor cocktail (Sigma-Aldrich). Cell lysates were subjected to 15% SDS-PAGE and immunoblotting as described [8]. Anti-Nef (1∶500) and human HIV-1 immunoglobulin (1∶3,000, both from the NIH Research and Reference AIDS Reagent Program) were used as primary antibodies. Horseradish peroxidase-conjugated anti-mouse IgG (1∶5,000, Promega) or anti-human IgG (1∶25,000, Promega) were used as secondary antibodies respectively. Restore Western blot stripping buffer (Pierce) was used to strip antibodies from probed membranes. Super-Signal chemiluminescence substrates (Pierce) were used to detect secondary antibodies.

### Flow cytometry

Cells (1×10^5^) were stained with specific monoclonal antibodies or isotype-matched IgG controls (2 µg/ml) as previously described [9]. Phycoerythrin (PE)- or fluorescein isothiocyanate (FITC)-conjugated mouse anti-human monoclonal antibodies against the following molecules were used in immunostaining: CD4 (clone S3.5; Caltag Laboratories), DC-specific intercellular adhesion molecule-3-grabbing non-integrin (DC-SIGN, clone number 120507; R&D Systems), CD86 (clone number BU63; Invitrogen). Negative controls were antibodies matched for isotype and fluorescent conjugation: mouse IgG_2a_ (PE-conjugate; BD Biosciences), mouse IgG_2_ (FITC-conjugate; BD Biosciences) or mouse IgG_1_ (FITC-conjugate; Invitrogen) respectively. Stained cells were analyzed using a FACSCalibur or a Guava EasyCyte flow cytometer (Millipore) and data was analyzed using the CellQuest program (Becton Dickinson) or FlowJo software (Tree Star) as previously described [12].

### HIV-1 infection and transmission assays

HIV-1 transmission and infection assays using luciferase viruses were performed as described previously [20]. Cell lysates were obtained 2 days after infection and analyzed for luciferase activity with a commercially available kit (Promega). For DC infection and transmission assays using replication-competent HIV-1, DCs (2.5×10^5^) were incubated separately with WT and Nef-mutated HIV-1 (50 ng p24) for 16 h. Cells were then washed thoroughly and cultured for the indicated time course. Cell-free supernatants from the HIV-1-infected DCs were harvested for Gag p24 quantification by ELISA at the indicated times post-infection. Resting CD4^+^ T cells (3×10^5^) were infected with 5 ng p24 of HIV-1_NLAD8_ WT or Nef-mutated viruses, or a media control, for 2 hours at 37°C, washed once in PBS and then cultured for 3, 5 or 7 days. CD4^+^ T cells were resuspended at 5×10^5^ cells/ml and added directly to the infected DCs at a ratio of 1∶1. HIV-1 p24 levels in the supernatants of infected cells were measured by ELISA at the indicated times.

### Assessment of CD69 expression as a marker of CD4^+^ T cell activation

Cells were harvested 3 dpi and fixed in 2% paraformaldehyde for 1 hour at 4°C. The fixed cells were stained with a PE-conjugated antibody to CD69 (clone number FN51; BD Biosciences) and a FITC-conjugated antibody to CD3 (clone number HIT3a; BD Biosciences). Cells were assessed by flow cytometry using a Guava EasyCyte Mini and the percentage cells CD69 positive was measured. In DC-T cell co-cultures, cells were first analyzed for CD3 expression, then the highly CD3-positive T cells were gated and analyzed for CD69 expression.

### CD4^+^ T cell proliferation assay

Purified resting CD4^+^ T cells were labeled with CFSE according to the manufacturer's instructions (Molecular Probes), infected and cultured or co-cultured with DCs as described above. At indicated time points, cells were harvested from culture and fixed in 2% paraformaldehyde for 1 hour at 4°C. Cells were assessed by flow cytometry using a Guava EasyCyte mini and proliferation was quantified using the proliferation analysis platform of the FLowJo flow cytometry software (Tree Star).

### Statistical analysis

Data were analyzed using a two-way ANOVA test and Bonferroni post-test or non-parametric ANOVA. Statistical significance was defined as *P*<0.05.
